# Epicardial adipose tissue and diastolic dysfunction: a relationship with unanswered questions

**DOI:** 10.1093/ehjimp/qyae072

**Published:** 2024-07-18

**Authors:** Stefano Nistri, Donato Mele

**Affiliations:** Department of Cardiac Thoracic Vascular Sciences and Public Health, University of Padua, Via Giustiniani, 2, 35128 Padua, Italy; Cardiology Service, CMSR Veneto Medica, Via Vicenza 204, 36077 Altavilla Vicentina (VI), Italy; Department of Cardiac Thoracic Vascular Sciences and Public Health, University of Padua, Via Giustiniani, 2, 35128 Padua, Italy

Epicardial adipose tissue (EAT) is the fat located between the myocardium and the visceral layer of the epicardium. Its relevance is not only due to its anatomy (i.e. proximity to myocardium and coronary arteries) but also to its distinctive trascriptome, which differentiates EAT from subcutaneous and other visceral fat depots. Even more intriguing, the regional distribution of EAT (i.e. atrial or pericoronary) has an important role because each EAT depot is anatomically, genetically, and functionally different. All these aspects have been elegantly reviewed by Iacobellis.^1^ The potential role of cardiovascular imaging has been ushered by the seminal echocardiographic studies by Iacobellis *et al*.^2,3^ more than 20 years ago. Since then, the interest in this field has exponentially grown with any available modality including echocardiography, computed tomography (CT),^18^F-FDG-PET–CT, and magnetic resonance. CT can measure EAT volume and thickness, regional EAT locations, assess pericardial adipose tissue, and estimate EAT density, which is of particular relevance also considering the increasing CT utilization in prevention and diagnosis.^1^ EAT has been linked to a variety of abnormal cardiovascular conditions including coronary artery disease, left ventricular diastolic dysfunction (LVDD), heart failure with preserved ejection fraction, and atrial fibrillation.^1^ Of interest, EAT is associated with diastolic function, independent of other influential variables and is an effect modifier for chamber size but not systolic function.^4^

In this issue of EHJ-IMP, Ishikawa *et al*.^5^ describe their retrospective, observational experience in 314 symptomatic individuals [66 ± 13 years (range: 40–85 years), 52% males] with chronic coronary syndrome (CCS), normal LVEF, in sinus rhythm, with no asinergy, without previous coronary revascularization, previous cardiac surgery or significant valvular heart disease, undergoing a comprehensive transthoracic echocardiogram 2 weeks before a coronary CT angiography. Their study was aimed at evaluating the relationships between EAT volume, coronary artery disease, and LVDD. EAT volume was classified as normal (<68.1 cm^3^/m^2^), low (68.1–89.4 cm^3^/m^2^), and high (>89.4 cm^3^/m^2^). LV diastolic function was described as normal (LVDD(−)), indeterminate or abnormal (LVDD(+)) based on the algorithm A of current guidelines. EAT was normal in 58% of patients displaying LVDD(-), but was abnormal virtually in all patients with LVDD(+), and in the vast majority of those with undetermined LVDD. Importantly, normal LV diastolic function was present—though decreasingly—in a significant proportion of patients with low or high EAT volume index, respectively. Consistently with previous findings, all the parameters used to assess LVDD were related to EAT volume index but tricuspid valve regurgitant peak velocity. EAT was also associated to LV mass index and, as a novel contribution, correlated significantly with left atrial volume index over a′ ratio (LAVI/a′). Noteworthy, EAT volume index and age were independently associated with LVDD even after adjusting for LV mass index, while LVDD was not significantly related to coronary artery calcium score or plaque volume.

The authors have to be congratulated for their efforts to provide a further contribution showing the correlation between EAT and LVDD, beyond age and LV mass. Their findings add interest in this topic but some limitations should be underscored.

Patients were labelled as ‘symptomatic’ with CCS. It is not clear which kind of symptoms the authors are talking about: did patients experience chest pain? Or shortness of breath? Was ischaemia objectively assessed in each patient? Indeed, only a minority of patients had obstructive coronary artery disease, a common finding in subjects with myocardial ischaemia in clinical practice underscoring the importance of coronary microvascular disease (CMD).^6^ If shortness of breath was the leading symptom, the potential role of exercise-induced LVDD (isolated or combined with myocardial ischaemia) should have been considered too. Whether these two different ‘symptom-phenotypes’ relate to different ‘EAT-phenotype’ is left unanswered by the present study.

Diastolic function was assessed by algorithm A of current guidelines, based on normal LVEF and absence of asinergy in the inclusion criteria. However, the utilization of this algorithm is debateable, since it is likely that a proportion of patients in this cohort might have had increased LV hypertrophy. It should be sincerely recognized, however, that this approach is frequently used in clinical practice, and that such a ‘simplified’ algorithm has been utilized in multiple studies.^7^ Anyway, the dichotomy of LVDD+/− raise some concerns. Diastolic dysfunction encompasses a range of severity including different LV filling pressures portending progressively worse expected outcomes. Thus, an appropriate grading should have been provided.^7,8^

As expected, a significant proportion of patients were classified as undetermined LVDD.^8–10^ Recently, patients with indeterminate diastolic function were shown to have higher risk of cardiovascular death or admission for HF than those with LVDD(−). Presence of CMD and elevated LV filling pressure were independent predictors for cardiovascular death or admission for HF among patients with indeterminate diastolic function.^9^ Pre-clinical atrial dysfunction is characterized by reduced LA reservoir and conduit function, while atrial contractile function remains normal. Consistently, assessment of LA function [mostly by speckle-tracking echocardiography (STE)] is now considered to be of interest when assessing indeterminate LVDD.^10^ However, STE is not widely available, particularly for the assessment of LA function, in many clinical settings. As late-diastolic a′ by tissue Doppler echocardiography reduces mirroring further deterioration of LV compliance, while LAVI progressively increases, LAVI/a′ is a likely candidate for detecting raised LV end-diastolic pressure when STE is not available.^8^ Indeed, as a novel contribution, the authors show that EAT volume index correlated significantly with LAVI/a′. Regrettably, though previous studies demonstrated that LAVI/a′ can sort undetermined LVDD,^8^ the authors did not take the opportunity to utilize this parameter to entangle indeterminate LVDD. Overall, these limitations do not consent to obtain a more granular picture of the correlation between EAT and different degrees of LVDD.

Finally, no data are available in this research, as in many other studies, regarding the regionality of EAT. EAT has emerged as a risk factor and independent predictor of atrial fibrillation development and recurrence after ablation.^11,12^ An association has also been reported between EAT volume or thickness and atrial conduction delays such as prolonged P-wave duration, interatrial conduction block and longer P–R interval.^13^ Thus, the present data do not help us understanding whether EAT affects LVDD by different regional (e.g. atrial vs. pericoronary) mechanism flawing their translation into clinical practice. Considering the expanding utilization of CT for preventive (i.e. calcium score) and diagnostic (i.e. coronary angiography) purposes, increasing interest in EAT is relevant both for research and clinical purposes; also considering the potential for appropriate therapeutical interventions.^1^ The paper by Ishikawa *et al*. is thus welcome to spread this message throughout the clinical community and to incite properly designed prospective studies to respond many of these unanswered questions.

**Conflict of interest:** None declared.

## Data Availability

No new data were generated or analysed in support of this editorial comment.

## Lead author biography

Stefano Nistri, MD, PhD, took his Degree in Medicine at Florence University, and his Postgraduate in Cardiology at Padua University. Thereafter, he completed his PhD in Experimental and Applied Internal Medicine at Florence University. His main interests focus on the utilization of echocardiography in the assessment of valvular heart disease, on hypertrophic cardiomyopathy aortopathies, diastolic function, and in translational cardiology, focusing on primary care outpatient settings. He served as a fellow in the Cardiomyopathy Center at Careggi Florence Hospital and, subsequently, in the Marfan Center of the same hospital. It is actually charged with a research programme at the Department of Cardiac, Thoracic, Vascular Sciences and Public Health of Padua University. He carries his clinical activity in a primary care outpatient setting, accredited with the Italian National Health System, as Chief of the Cardiology Service of CMSR Veneto Medica in Altavilla Vicentina, Italy.
